# Prominent urinary dysfunction and delayed antibody detection in patients with glial fibrillary acidic protein astrocytopathy: a case series analysis

**DOI:** 10.1186/s12883-025-04606-6

**Published:** 2026-01-02

**Authors:** Zhenyu Niu, Yunchuang Sun, Fan Li, Zhaoxia Wang, Luhua Wei, Ran Liu, Feng Gao, Haiqiang Jin, Jing Guo, Nan Zhang, Yiming Zheng, Hongjun Hao

**Affiliations:** 1 Department of Neurology, Peking University First Hospital, 8 Xishiku Street, Xicheng District, Beijing, 100034 China; 2 Rare Disease Medical Center, Peking University First Hospital, 8 Xishiku Street, Xicheng District, Beijing, 100034 China

**Keywords:** Glial fibrillary acidic protein, Autoimmune diseases of the nervous system, Central nervous system, Urinary retention, Autonomic dysfunction

## Abstract

**Background:**

Glial fibrillary acidic protein astrocytopathy (GFAP-A) is a rare central nervous system (CNS) autoimmune disorder with highly heterogeneous presentations. This study aims to describe the clinical and diagnostic features in a Chinese case series.

**Methods:**

Six patients at a single tertiary center with positive GFAP antibody were included, the patients’ data were retrospectively analyzed.

**Results:**

The cohort included 3 males and 3 females, with a median age of 56 years (range 30–75). Prominent urinary dysfunction was observed in four patients (66.7%). Brain or spinal cord MRI abnormalities were present in only 50% (3/6) of patients. All patients exhibited CSF-specific oligoclonal bands and elevated indices of intrathecal IgG synthesis. A notable finding was the delayed detection of CSF GFAP antibody in five patients (83.3%), who tested negative at disease onset but turned positive upon follow-up testing weeks to months later.

**Conclusion:**

This case series underscores the high frequency of autonomic urinary dysfunction and the potential for delayed antibody detection in Chinese GFAP-A patients. These findings suggest that clinical suspicion should remain high even with initial negative antibody testing, warranting repeat CSF analysis in suspected cases to avoid diagnostic delay.

**Supplementary Information:**

The online version contains supplementary material available at 10.1186/s12883-025-04606-6.

## Introduction

Autoimmune glial fibrillary acidic protein astrocytopathy (GFAP-A), recognized since 2016 [1], is a central nervous system (CNS) autoimmune disorder characterized by immunoglobulin G (IgG) antibody against GFAP, an astrocyte-specific cytosolic protein [2]. GFAP-A includes various symptoms like encephalopathy, optic papillitis and myelitis, often accompanied by linear perivascular enhancements on magnetic resonance imaging (MRI) with inflammatory changes in cerebrospinal fluid (CSF) [3]. Detection of GFAP-specific IgG antibody in body fluids, particularly in CSF, is the primary diagnostic criterion for GFAP-A, while treatment with corticosteroids is effective but often requires ongoing immunosuppression due to frequent relapses.

For clinicians, diagnosing GFAP-A is often challenging due to its variable presentation, the absence of definitive non-antibody criteria, and the limited sensitivity of MRI. Brain and spinal cord MRI abnormalities are respectively observed in only 50–80% and 49–66% of patients [4–6], and not all of whom exhibit the classic linear perivascular enhancements. Moreover, common CSF changes are indistinguishable from other encephalitis, while the high frequency of co-existing autoimmunity or malignancies complicates the attribution of symptoms solely to GFAP antibody [7].

In this article, we retrospectively analyzed six patients with GFAP-A, five of whom exhibited prominent autonomic dysfunction, notably refractory urinary symptoms. Furthermore, these five patients demonstrated “delayed antibody detection,” testing negative at onset but seroconverting upon follow-up testing weeks to months later. This case series highlights urinary dysfunction and delayed antibody positivity as key clinical features, suggesting that repeated antibody testing may be necessary in suspicious cases, ultimately aiming to enhance early diagnosis and management.

## Methods

### Patients

Six patients admitted to the Department of Neurology at Peking University First Hospital were included in the retrospective study, tested positive for CSF GFAP antibody between May 2021 and June 2024. Paired serum and CSF samples were collected from each patient for analysis. All exhibited symptoms affecting the meninges, spinal cord, spinal nerve roots, or autonomic nervous system. These clinical findings confirmed their diagnosis of GFAP-A. This study is a retrospective analysis and has obtained the consent of each patient. All of the research procedures involving human were in accordance with the Declaration of Helsinki. The study was approved by the institutional review boards of Peking University First Hospital Ethics Committee.

### Examination of GFAP antibody

For each test, patients provided paired serum and CSF samples. The process involved cell-based assays (CBA), using the HEK293 cell line transfected with the GFAP gene. Transfected and control cell slides were fixed in distinct reaction zones on a microscope slide. Following the addition of gradient-diluted serum and CSF samples, the reaction proceeded to completion. The slides were then washed, and fluorescein isothiocyanate-labeled anti-human IgG antibodies were applied. After adequate reaction time and subsequent washing, the slides were examined under a microscope. A test was considered positive when specific green fluorescence was observed on transfected cells at a serum dilution of ≥ 1:10 or a CSF dilution of ≥ 1:1, with no fluorescence on control cells.

### Other neuroimmunology tests

Immunofixation electrophoresis, combined with immunofixation, was utilized to identify oligoclonal bands (OCB) in six enrolled patients’ paired serum and CSF. Cell-based assays were employed to detect aquaporin-4 (AQP4) and myelin oligodendrocyte glycoprotein (MOG) antibodies in serum and CSF. Additionally, certain patients underwent cell-based assays for the autoimmune encephalitis antibody panel, which includes α-amino-3-hydroxy-5-methyl-4-isoxazole-propionic acid receptor (AMPAR), N-methyl-D-aspartic acid receptor (NMDAR), and dipeptidyl-peptidase-like protein 6 (DPPX) antibodies. During each test, albumin and IgG levels in serum and CSF were measured to calculate the albumin index, IgG index, and the 24-hour IgG synthesis rate.

## Results

### Demographic and clinical profiles of six patients

The study involved six Han Chinese patients (3 males, 3 females) with onset ages ranging from 30 to 75 years. None had a history of neoplasm.

The initial symptoms commonly included persistent fever (83.3%, 5/6) and severe headache (66.7%, 4/6). As the disease progressed, symptoms of disorientation, impaired comprehension, and disturbances in consciousness emerged in half of the patients (50.0%, 3/6). Brainstem and cerebellar involvement was observed in two patients (33.3%), presenting as significant ataxia in one and dysphagia in the other. Weakness, predominantly in the bilateral lower limbs, was frequent (66.7%, 4/6) and was sometimes accompanied by numbness or muscle pain. Additionally, a majority of patients (66.7%, 4/6) experienced persistent dysuria and constipation. Patient 6 presented a distinct clinical profile characterized by rapidly progressive dementia and frequent seizures of frontal lobe origin; physical examination findings were consistent with cortical involvement (Table 1).

**Table 1:** Patients’ demographic, 3T-MRI and clinical features

No	Sex	Age (years)	Symptoms	Locations by Physical Examination	MRI Features	OCB	GFAP Antibody		Other CSF and Serum Autoimmune Antibodies
							CSF (CBA)	Blood (CBA)	
1	M	56	Headache; Fever; DoC; Delirium; Psychosis; Weakness	Meninges; Cortex; Spinal cord	Brain: Normal Spinal cord: Normal	Type III	Positive(1:20)	Negative	Negative
2	F	30	Headache; Fever; Syncope; Dysuria; Leg Muscle Pain	Meninges; Autonomic nerve; Spinal nerve roots; Motor nerve	Thick ventral pia cranialis of both Brain and Spinal cord T1-weighted enhance	Type II	Positive(1:5)	Positive(1:10)	Negative
3	F	59	Ataxia; Fever; Numbness; Weakness; Dysuria; Constipation	Brainstem; Spinal cord; Autonomic nerve; Spinal nerve roots	Multiple lesions and T1-weighted enhance in subcortical white matter, brainstem and spinal cord	Type II	Positive(1:1)	Negative	Negative
4	M	59	Headache; Fever; Hyperalgesia; Weakness; Dysuria; Constipation	Meninges; Spinal cord; Autonomic nerve	Brain: Normal Spinal cord: Normal	Type II	Positive(1:10)	Negative	Negative
5	M	75	Headache; Fever; DoC; Dysphagia; Weakness; Dysuria	Meninges; Cortex; Brainstem; Spinal cord; Autonomic nerve; Spinal nerve roots	Brain: Normal Spinal cord: Normal	Type II	Positive(1:20)	Negative	Negative
6	F	51	Dementia; DoC; Epilepsy	Cortex	T1-weighted enhance in cranialis, brainstem and cerebellum Spinal cord: Normal	Type III	Positive(1:40)	Positive(1:160)	Negative

*DoC* disorders of consciousness, *OCB* oligoclonal bands, *CSF* cerebrospinal fluid, *CBA* cell based assays

### MRI features of patients

**Fig. 1:**
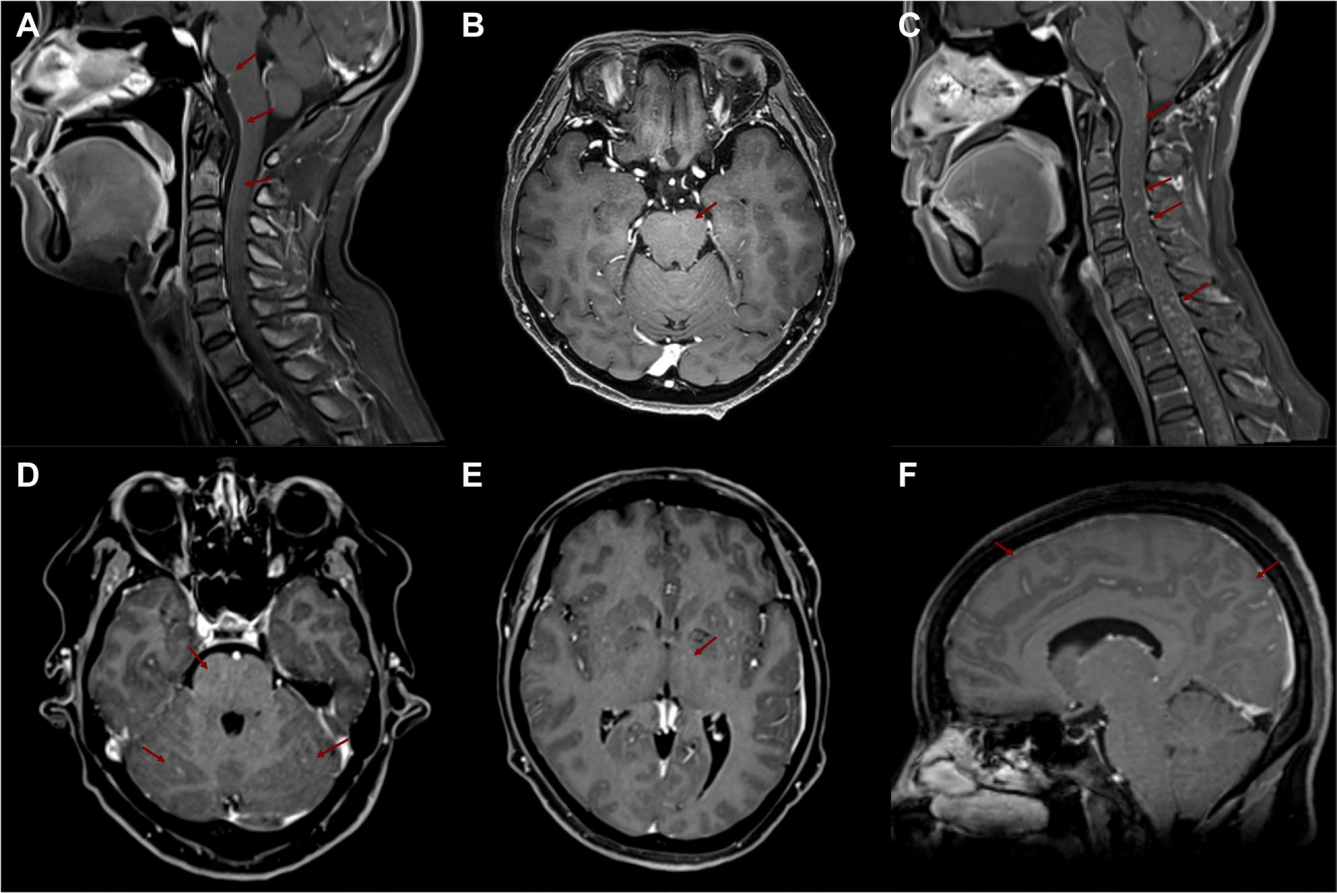
Brain and spinal cord MRI findings in three GFAP-A patients with abnormal imaging. **A** Patient 2: T1-weighted contrast-enhanced image showing leptomeningeal enhancement along the brainstem and ventral surface of the spinal cord (arrows). **B**, **C** Patient 3: Axial and sagittal T1-weighted contrast-enhanced images demonstrating multiple punctate and patchy enhancements within the brainstem and cervical spinal cord (arrows). **D**, **E**, **F** Patient 6: T1-weighted contrast-enhanced images revealing punctate and linear enhancements in the corona radiata, basal ganglia, brainstem, and cerebellum, accompanied by diffuse leptomeningeal enhancement along the cerebral sulci (arrows)

Brain and entire spinal cord MRI scans, including T1-weighted enhanced sequences at 3T, were performed in all six patients (Table 1). Imaging abnormalities were detected in 50% (3/6) of patients. All patients with positive findings demonstrated abnormal enhancement on post-contrast T1-weighted sequences. Patient 3 exhibited multifocal enhancing lesions in the subcortical white matter, brainstem, and spinal cord (Fig. 1B, C). Patient 2 showed cranial and spinal leptomeningeal enhancement (Fig. 1A), while Patient 6 had enhancing lesions located in the brainstem and cerebellum (Fig. 1D, E, F).

### Routine CSF analysis and autoimmune antibodies of patients

**Fig. 2:**
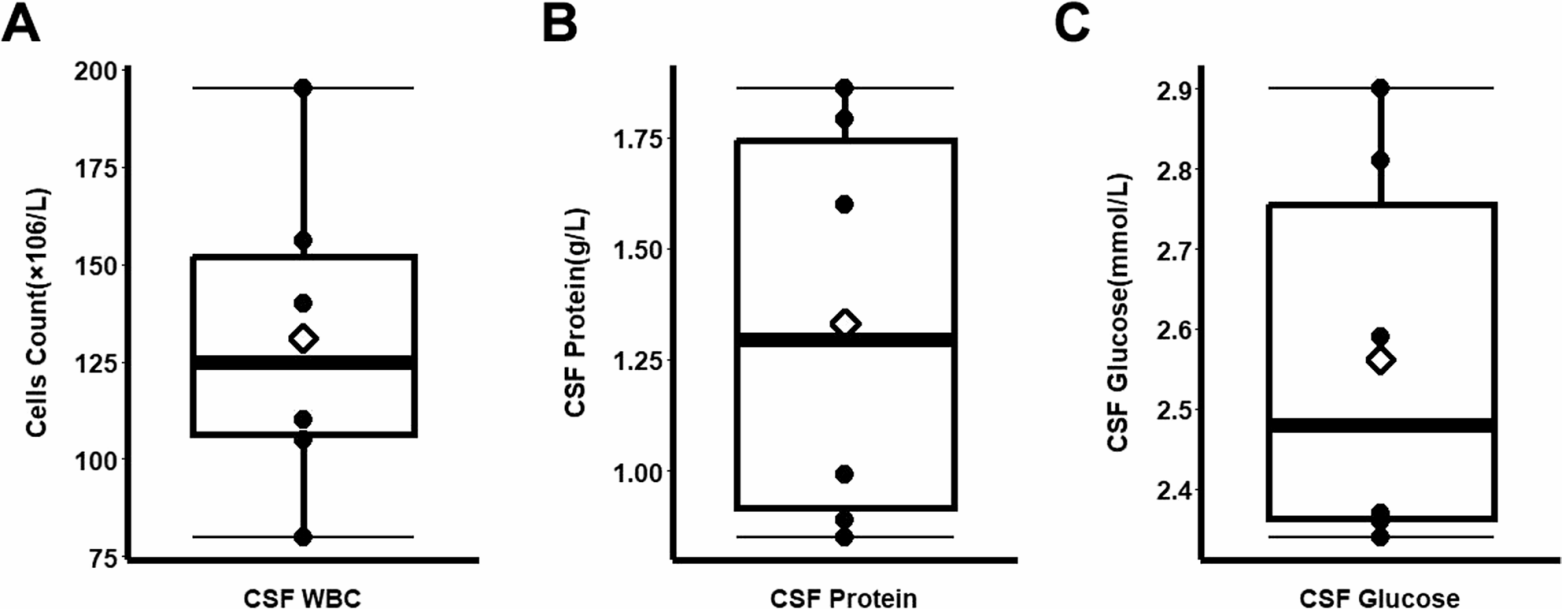
CSF routine and biochemical profiles at initial presentation. Box plots (median, quartiles) illustrating the initial CSF findings across the six patients: **A** White blood cell (WBC) count, **B** Protein concentration, and **C** Glucose level. *Note*: CSF, cerebrospinal fluid; WBC, white blood cells

CSF analysis was performed via lumbar puncture on all six patients during initial hospitalization. The median CSF white blood cell count was 107.5 × 10⁶/L (Q1: 92.5, Q3: 148) (Fig. 2A). Elevated CSF protein levels were observed, with a median of 1.25 g/L (Q1: 0.92, Q3: 1.63) (Fig. 2B). The median CSF glucose level was 2.48 mmol/L (Q1: 2.36, Q3: 2.77) (Fig. 2C). All patients tested positive for CSF-specific OCB. Four patients had OCB restricted to the CSF (Type II), while Patients 2 and 6 had matching OCB bands in both CSF and serum (Type III) (Table 1).

All six patients were positive for CSF GFAP antibody, with titers ranging from 1:1 to 1:160. Serum GFAP antibody was concurrently positive in Patients 2 and 6. No other autoantibodies, including AQP4 antibody, MOG antibody, neuronal surface/onconeural antibodies, or thyroid antibodies, were detected. In five patients (all except Patient 6), the initial CSF GFAP antibody test was negative, with seroconversion occurring on subsequent testing. The time from symptom onset to a positive GFAP antibody result was up to three months in two patients (Table 2).

**Table 2:** Temporal profile of CSF GFAP antibody detection in six patients

No	CSF GFAP Antibody (CBA) after Onset										
	<1w	2w	3w	1m	5w	6w	2m	3m	4m	6m	1y
1	(-)	(-)		(+)		(+)					(+)
2	(-)		(+)	(+)							(-)
3							(-)	(+)	(+)		(-)
4		(-)	(-)	(-)			(+)		(-)		
5		(-)	(-)	(-)	(-)	(-)		(+)			(+)
6				(+)						(-)	

*CSF* cerebrospinal fluid, *CBA* cell based assays, *W* week, *M* month, *Y* year

### Other CSF examinations of patients

**Fig. 3:**
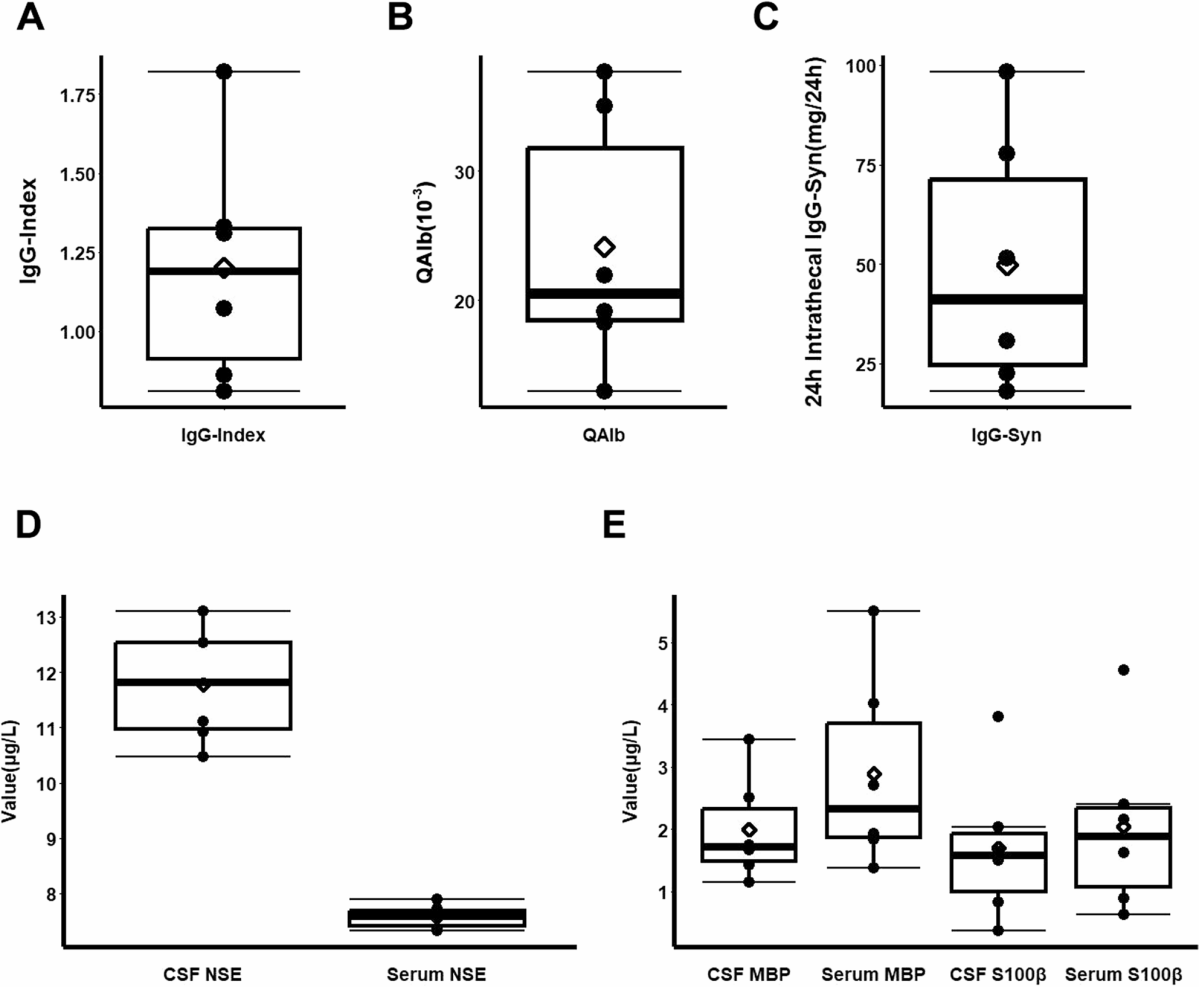
CSF immunological and injury biomarker profiles. **A**-**C** Box plots (median, quartiles) displaying parameters of intrathecal immune response: IgG Index (**A**), CSF/Serum Albumin Ratio (**B**), and 24-hour IgG Synthesis Rate (**C**). **D**, **E** Levels of central nervous system injury biomarkers in paired CSF and serum samples: Neuron-Specific Enolase (**D**), Myelin Basic Protein and S100β (**E**). Data are presented as box plots (median, quartiles). *Note*: IgG, Immunoglobulin G; QAlb, cerebrospinal fluid/serum albumin ratio; IgG-Syn, The 24-hour IgG synthesis rate by Tourtellotte equation; CSF, cerebrospinal fluid; NSE, neuron specific enolase; MBP, myelin basic protein; S100β, central nervous system specific protein β

Paired CSF and serum samples were analyzed for albumin and IgG. The median CSF albumin index was 22.54 × 10⁻³ (Q1: 17.38, Q3: 29.21). The median 24-hour IgG synthesis rate was 46.11 mg/24 h (Q1: 26.39, Q3: 73.52). The median IgG Index was 1.07 (Q1: 0.92, Q3: 1.31) (Fig. 3A-C).

Levels of neuron-specific enolase (NSE), myelin basic protein (MBP), and S100β were measured in paired CSF and serum as biomarkers of CNS injury. The median CSF NSE level was 11.82 µg/L (Q1: 10.87, Q3: 12.53), was significantly higher than normal populations. While serum NSE, as well as both CSF and serum levels of MBP and S100β, remained generally normal (Fig. 3D and E).

### Treatment of patients

All six patients received steroid therapy. Five patients (excluding Patient 5) underwent high-dose methylprednisolone pulse therapy following GFAP antibody confirmation. Patients 1, 3, 4, and 6 started at 1000 mg daily, while Patient 2 began at 500 mg daily due to milder symptoms. These five followed a standard taper, halving the dose every three days to 125 mg, then transitioning to 60 mg of oral prednisone with gradual reduction.

Patient 5, initially seronegative, started oral prednisone at 20 mg/day one month after onset due to poor response to antivirals. After subsequent antibody detection, the dose was increased to 50 mg/day and tapered by 5 mg weekly to 25 mg, then by 5 mg every two weeks.

Due to severe symptoms and delayed diagnosis, Patients 1 and 5 received intravenous immunoglobulin (IVIg; 0.4 g/kg/day for 5 days) before steroid initiation and required ICU care. Patient 6, presenting with fever, impaired consciousness, and status epilepticus, underwent three plasma exchange sessions for rapid stabilization.

### Outcome and follow-up of patients

All six patients responded well to corticosteroid treatment, with no relapses observed during the follow-up period. Patients were followed for a median of 13.5 months (range: 8–21). The detailed follow-up data are summarized in Supplemental Table S1. At the last follow-up, the majority of patients (4/6, 66.7%) had a modified Rankin Scale (mRS) score of 0–1 [8]. The most common residual symptom was bladder dysfunction (50.0%), followed by motor symptoms (33.3%).

Follow-up MRI of the brain, performed at a median of 4 months, showed improvement in two of the three patients with initial abnormalities, with the remainder stabilized. All spinal cord MRIs, including two patients with initial abnormal scan, remained stable on follow-up. Follow-up CSF analysis was conducted at a median of 9.5 months. Normalization of CSF white cell count and protein level was observed in 100% and 83.3% of patients. CSF GFAP antibody turned negative in four of the six patients (66.7%), with the exception of Patients 1 and 5—both of whom had severe initial symptoms and received IVIg—who remained positive. In Patients 1 and 5, no significant change was found in antibody titer levels (Table 2). The two patients with initially positive serum GFAP antibody both converted to negative.

No relapses occurred during the follow-up period. The majority of patients (83.3%) received oral steroid therapy for more than three months. No patients required long-term treatment with IVIg, rituximab, or other oral immunosuppressants.

## Discussion

In this case series of six Chinese patients with GFAP-A, we highlight two salient clinical-diagnostic features: a high prevalence of prominent autonomic dysfunction, particularly urinary retention, and a consistent pattern of delayed antibody detection in the CSF. These observations, when contextualized with recent literature, have significant implications for the clinical recognition, pathophysiological understanding, and management of this autoimmune astrocytopathy.

The burden of autonomic nerve involvement in our cohort was substantial. Four of the six patients (66.7%) experienced significant and often refractory urinary dysfunction. This aligns with a growing body of evidence suggesting that autonomic dysfunction, especially bladder and gastrointestinal involvement, is one of the core features of GFAP-A. A recent case series focusing on autonomic dysfunction in GFAP-A reported an astonishingly high prevalence of bladder dysfunction (97.6%) [9], indicating that our findings may still represent a conservative estimate. The persistence of these symptoms in our patients, particularly the refractory urinary retention in Patient 5, suggests a specific vulnerability of the autonomic pathways within the spinal cord (e.g., the conus medullaris or the autonomic nuclei) [9]. This is further supported by the high frequency of spinal cord involvement (5/6 patients) in our series and the observation of spinal leptomeningeal enhancement, a feature recently emphasized in Chinese cohorts [4, 10].

The dissociation between pronounced clinical symptoms and the presence of MRI abnormalities in only half of our patients highlights the limited sensitivity of conventional MRI in this condition, a finding consistent with larger meta-analyses [6]. The universal finding of T1-weighted post-contrast enhancement among MRI-positive cases is a key radiological feature, indicating active inflammation associated with blood-brain barrier disruption.The imaging pattern observed in Patient 3, with multifocal enhancing lesions in the subcortical white matter, brainstem, and spinal cord, bears a notable resemblance to Chronic Lymphocytic Inflammation with Pontine Perivascular Enhancement Responsive to Steroids (CLIPPERS) syndrome. This similarity may offer insights into shared immunopathological mechanisms or specific anatomical targets of inflammation.

A second critical finding was the phenomenon of delayed antibody detection. In five of our six patients, the initial CSF GFAP antibody test was negative, with conversion occurring weeks to months after symptom onset, which presents a significant diagnostic challenge in the early disease phase. This delayed antibody detection may reflect the time required for a T-cell-dependent initiation and maturation of a specific autoimmune response within the CNS. While this delayed positivity has not been a major focus in large cohort studies [6, 11], its high frequency in our small series underscores a potential diagnostic pitfall. Our findings strongly suggest that a single negative GFAP antibody test, particularly early in the disease course, cannot definitively exclude the diagnosis. The increased CSF cells and protein, coupled with the universal evidence of intrathecal immunoglobulin synthesis (as demonstrated by the presence of types II and III OCB), collectively provide strong support for a robust, compartmentalized humoral immune response within the CNS. The clinical implication is clear: in those patients with a high pre-test probability of GFAP-A—based on symptoms of meningoencephalomyelitis, prominent autonomic dysfunction, or suggestive MRI findings—repeated CSF and serum antibody testing is warranted. This practice could prevent diagnostic delay and ensure timely initiation of immunotherapy.

Beyond these core findings, our cohort reflected the known heterogeneity of GFAP-A. Patient 6 presented with rapidly progressive dementia and epilepsy, an atypical phenotype that expands the clinical spectrum and underscores the importance of including GFAP-A in the differential diagnosis of cognitive decline, as also suggested by case reports from other populations [11–13]. In addition, the favorable response to steroid in our entire cohort is consistent with the high response rates (77–83%) reported in the literature [4, 6].

The exact pathophysiological role of GFAP antibody remains enigmatic. The observed delay in antibody appearance, coupled with the fact that GFAP is an intracellular antigen, supports the hypothesis that the antibody production might be a secondary, epiphenomenal response to astrocytic damage rather than the primary cause [4]. The initial injury could be triggered by a viral infection [14] or a T-cell-mediated attack, with recent evidence highlighting the role of CD8 ^+^ T cells [4, 15]. The fact that Patient 5 converted after steroid treatment, during clinical improvement, further challenges a purely primary pathogenic role for the antibody and warrants further investigation. Our finding that urinary dysfunction was a common residual symptom is consistent with long-term follow-up studies which show that, despite good overall functional outcomes (mRS 0–1), a significant proportion of patients are left with disabling sequelae, including bladder dysfunction [9, 11].

Our study has several limitations. Its retrospective design and small sample size from a single center limit the generalizability of our findings and preclude definitive statistical conclusions. The treatment regimens were not uniform across all patients, which may have influenced individual outcomes. Finally, while our follow-up duration was informative, a longer observation period would be required to fully understand the long-term prognosis and relapse risk, especially given that relapses can occur months after onset. Future prospective, multi-center studies with larger cohorts are needed to validate our observations regarding urinary dysfunction and delayed antibody detection.

## Conclusion

In summary, our case series, in concert with recent literature, highlights prominent urinary dysfunction and delayed antibody detection as key features in GFAP-A. These findings suggest that clinicians should maintain a high index of suspicion for GFAP-A in patients with compatible CNS inflammation and autonomic symptoms, and should not be deterred by an initially negative antibody test. Repeated testing and early empirical immunotherapy may be justified in highly suggestive cases. Further research is needed to elucidate the mechanisms underlying the observed autonomic predominance and the delayed humoral immune response in this intriguing autoimmune disorder.

## Supplementary Information

Supplementary Material 1.

## Data Availability

All data analyzed during this study are available from the corresponding author upon reasonable request.

## Abbreviations

Abbreviation: Definition
AMPAR: α-amino-3-hydroxy-5-methyl-4-isoxazole-propionic Acid Receptor
AQP4: Aquaporin-4
CBA: Cell-based Assays
CLIPPERS: Chronic Lymphocytic Inflammation with Pontine Perivascular Enhancement Responsive to Steroids Syndrome
CNS: Central Nervous System
CSF: Cerebrospinal Fluid
DPPX: Dipeptidyl-peptidase-like Protein 6
GFAP: Glial Fibrillary Acidic Protein
GFAP-A: Glial Fibrillary Acidic Protein Astrocytopathy
IgG: Immunoglobulin G
IVIg: Intravenous Immunoglobulin
MBP: Myelin Basic Protein
MOG: Myelin Oligodendrocyte Glycoprotein
MRI: Magnetic Resonance Imaging
NMDAR: N-methyl-D-aspartic Acid Receptor
NSE: Neuron Specific Enolase
OCB: Oligoclonal Bands
S100β: Central Nervous System Specific Protein β
WBC: White Blood Cell
