# Supplementary material for: Prominent urinary dysfunction and delayed antibody detection in patients with glial fibrillary acidic protein astrocytopathy: a case series analysis
Source: BMC Neurol. 2026 Jan 2;26:63. doi: 10.1186/s12883-025-04606-6 (PMC12866294; doi:10.1186/s12883-025-04606-6)
Supplement: Supplementary file 1 — Supplementary Material 1. [file 12883_2025_4606_MOESM1_ESM.docx]

**Supplemental Table S1 Patients’ Long Time Follow up**

|  | Patients at Last Follow-up  (*N*=6) |
| --- | --- |
| Follow-up time (months), median (range) | 13.5 (8-21) |
| mRS, *n*(%) |  |
| 0 | 2 (33.3) |
| 1 | 3 (50) |
| 2 | 1 (16.7) |
| Disability, *n*(%) |  |
| Cognitive impairment | 0 (0) |
| Gastrointestinal dysfunction | 1 (16.7) |
| Bladder dysfunction | 3 (50) |
| Motor symptoms | 2 (33.3) |
| Sensory symptoms | 0 (0) |
| Time to follow-up MRI (months), median (range) | 4 (2-12) |
| Brain MRI, *n*(%) |  |
| Improvement | 2 (33.3) |
| Stabilization | 4 (66.7) |
| Worsening | 0 (0) |
| Spinal cord MRI, *n*(%) |  |
| Improvement | 0 (0) |
| Stabilization | 6 (100) |
| Worsening | 0 (0) |
| Time to follow-up CSF analysis (months), median (range) | 9.5 (4-12) |
| CSF analysis, *n*(%) |  |
| CSF protein normalization | 5 (83.3) |
| CSF white cell count normalization | 6 (100) |
| CSF OCB positive | 3 (50) |
| CSF GFAP antibody positive | 2 (33.3) |
| Serum GFAP antibody positive | 0 (0) |
| Relapse, *n* (%) | 0 (0) |
| Long-term treatment, *n* (%) |  |
| Oral corticosteroids >3 months | 5 (83.3) |
| IVIg >2 times | 0 (0) |
| Rituximab | 0 (0) |
| Oral Immunosuppressant | 0 (0) |
